# Numerical Investigation of Exergy Loss of Ammonia Addition in Hydrocarbon Diffusion Flames

**DOI:** 10.3390/e24070922

**Published:** 2022-07-01

**Authors:** Haifeng Sun, Zhongnong Zhang, Hanxiao Sun, Bin Yao, Chun Lou

**Affiliations:** 1 Huadian Electric Power Research Institute Co., Ltd., Hangzhou 310030, China; chdshf@126.com; 2 State Key Laboratory of Coal Combustion, School of Energy and Power Engineering, Huazhong University of Science and Technology, Wuhan 430074, China; 13971003680@163.com; 3 School of Engineering, University of Glasgow, Glasgow G12 8QQ, UK; 2698846s@student.gla.ac.uk

**Keywords:** ammonia, diffusion flame, entropy generation, exergy loss, thermodynamics efficiency

## Abstract

In this paper, a theoretical numerical analysis of the thermodynamics second law in ammonia/ethylene counter-flow diffusion flames is carried out. The combustion process, which includes heat and mass transfer, as well as a chemical reaction, is simulated based on a detailed chemical reaction model. Entropy generation and exergy loss due to various reasons in ammonia/ethylene and argon/ethylene flames are calculated. The effects of ammonia addition on the thermodynamics efficiency of combustion are investigated. Based on thermodynamics analysis, a parameter, the lowest emission of pollutant (LEP), is proposed to establish a relationship between the available work and pollutant emissions produced during the combustion process. Chemical reaction paths are also analyzed by combining the chemical entropy generation, and some important chemical reactions and substances are identified. The numerical results reveal that ammonia addition has a significant enhancement on heat transfer and chemical reaction in the flames, and the total exergy loss rate increases slightly at first and then decreases with an increase in ammonia concentration. Considering the factors of thermodynamic efficiency, the emissions of CO_2_ and NOx reach a maximum when ammonia concentration is near 10% and 30%, respectively. In terms of the chemical reaction path analysis, ammonia pyrolysis and nitrogen production increase significantly, while ethylene pyrolysis and carbon monoxide production decrease when ammonia is added to hydrocarbon diffusion flames.

## 1. Introduction

The utilization of ammonia, a hydrogen-based fuel, has been one of the most interesting issues in recent years. Previous works have demonstrated that adding ammonia to hydrocarbons reduces the formation of carbon dioxide and soot significantly [1,2,3,4,5,6,7,8]. Ammonia, on the other hand, has a higher power density and is easier to store and transport than hydrogen [9,10]. It is anticipated that the combustion method of incorporating ammonia into typical hydrocarbon fuel will have widespread application in the future of energy utilization systems, such as boilers and internal combustion engines. Additionally, from the view of energy utilization, the addition of ammonia has a substantial impact on the irreversibility of the combustion process [1]. It further results in a loss of available work, which has a significant relationship with the energy conversion efficiency [11]. Thermodynamics second-law analysis is an effective way of evaluating the ideal efficiency limit of working with various flames in this sector, and it has been one of the most interesting issues in recent years.

A systematic theoretical analysis approach of the thermodynamics second law has been developed, and it has been summarized by Som and Datta [12]. Entropy generation and exergy loss are two important parameters in the thermodynamics analysis. For the first time, Lior et al. [13] considered dividing the combustion process into several sub-processes; they analyzed the flame of hydrogen and hydrocarbon by a hypothetical approach and found that exergy destruction is mainly due to the internal heat exchange. Subsequently, the entropy generation is obtained based on the entropy conservation equation [14]. It is obtained from the irreversibilities of four sub-processes in the combustion process: viscous dissipation, heat conduction, mass diffusion, and chemical reaction. In addition, exergy loss, indicating loss of available work, can be calculated according to Gouy–Stodola equation. Han et al. [15] thought that, in energy utilization systems, exergy carried by incomplete combustion products will not be utilized and it should also be considered as a kind of exergy loss.

Furthermore, many works about the thermodynamic analysis of various flames and combustion systems have been reported throughout the last few decades. Nishida et al. [16] analyzed the entropy generation in one-dimensional premixed flames. They investigated the effects of inlet temperature and velocity of fuel, and the results showed that the entropy generation can be reduced by decreasing the inlet temperature of the fuel. Datta [17,18] calculated the entropy generation in a laminar jet diffusion flame, and he analyzed the effect of gravity on the rate of entropy generation. Meanwhile, Lou et al. [19,20,21,22,23] calculated the radiative entropy generation in flames, and they also investigated the effects of soot and temperature on entropy generation in hydrocarbon flames. Recently, the thermodynamics second-law analysis of the combustion process of hydrogen has received extensive attention. Mishra et al. [24] analyzed the effect of wall thermal conductivity on entropy generation and exergy losses in a hydrogen/air microcombustor. Yang et al. [25] studied the effect of a block insert on the entropy generation of a hydrogen microcombustor. The results revealed that the structure and structural parameters of the burner have an important influence on the irreversibility of the combustion process. In addition, scholars have conducted thermodynamic analyses on various combustion methods of hydrogen. The exergy loss and exergy loss ratio in syngas flames (H_2_/CO) were calculated and investigated, and the results revealed that increasing pressure, inlet temperature, and hydrogen concentration in fuel can reduce the exergy loss ratio of the flames [26,27]. Han et al. [28,29,30] developed a series of works about the thermodynamics analysis of combustion processes. The hydrogen flame, hydrogen/methane flame, and ammonia/hydrogen flame were investigated, as well as the effect of component concentration in the flame was analyzed.

It is worth noting that the thermodynamics second-law analysis has been extensively used in studies of the combustion process, focusing either on hydrogen flames or flames with a mixture of hydrogen and other fuels. However, the combustion method of ammonia has not been completely studied. Only Han et al. [30] developed a thermodynamic analysis of the ammonia/hydrogen premixed flame. Few works have been conducted to investigate the influence of ammonia addition on the thermodynamics efficiency of the combustion process in hydrocarbon flames. Therefore, in this work, the counter-flow diffusion flames with ammonia/ethylene and argon/ethylene fuel are considered. The exergy loss and exergy loss rate in various combustion processes are calculated. The effects of ammonia addition on exergy loss due to heat transfer, mass diffusion, chemical reaction, as well as incomplete combustion of the flames are investigated. The total exergy loss rate is given and the thermodynamics efficiency of various flames is evaluated. 

## 2. Methodology

### 2.1. Chemical Kinetic Modeling

The chemical mechanism in the combustion process of hydrocarbons and ammonia is complicated and related closely to the results of thermodynamics calculations. Previous work indicated that reducing mechanisms may lead to the inaccuracy of the calculation of entropy generation and exergy loss [27]. Therefore, a detailed NH_3_-C_2_H_4_-PAH combustion mechanism with 182 species and 1571 reactions was used for the numerical calculation of one-dimensional counter-flow diffusion flame [2]. The species considered in the kinetic is up to A4, and the oxidation process of hydrocarbons and ammonia, as well as the PAH formation process, are covered. The chemical mechanism has been validated based on the experimental results, including flame speed and mole fraction of components, in prior work [2].

### 2.2. Energy Generation Rate

According to the local equilibrium hypothesis and the Gibbs relation, the entropy balance equation can be given as:

(1) $$T\frac{ds}{dt}=\frac{du}{dt}+p\frac{d}{dt}\left(\frac{1}{\rho }\right)-\sum_{k}\mu_{k}\frac{d\omega_{k}}{dt}$$

where s is the specific entropy, u is internal energy, p is pressure, $\mu_{k}$ is the chemical potential of *k*th species and $\omega_{k}$ is the mass fraction of *k*th species.

For a multi-species, reacting mixture, the differential du/dt, dρ/dt, and $d\omega_{k}/dt$ in Equation (1) can be given as follows:

(2) $$\rho \frac{du}{dt}=-\nabla \cdot q-p\nabla \cdot v-\tau :\nabla v+\sum_{k}J_{k}\cdot F_{k}$$

(3) $$\frac{d\rho }{dt}=-\rho \nabla \cdot v$$

(4) $$\rho \frac{d\omega_{k}}{dt}=-\nabla \cdot J_{k}+r_{k}$$

where q and $J_{k}$ are the heat flux and the diffusive flux, respectively. v is the velocity vector, τ is the stress tensor, $F_{k}$ is a body force per unit mass and $r_{k}$ is the production rate of *k*th species.

Introducing Equations (2)–(4) into Equation (1), the entropy balance equation can be given as:

(5) $$\rho \frac{ds}{dt}=-\frac{1}{T}\nabla \cdot q-\frac{1}{T}\tau :\nabla v+\frac{1}{T}\sum_{k}\mu_{k}\nabla \cdot J_{k}-\frac{1}{T}\sum_{k}\mu_{k}r_{k}$$

Through Equation (5), the entropy generation rate can be further deduced, and the detailed derivation process has been provided in previous work [31]. The local entropy generation of combustion process are from the irreversibility of four sub-processes, which are thermal radiation, heat conduction, mass diffusion, chemical reaction, and viscous dissipation. These are given as:

(6) $$S_{c}=-\frac{1}{T^{2}}q\cdot \nabla T$$

(7) $$S_{\mu }=-\frac{1}{T}\tau :\nabla v$$

(8) $$S_{diff}=\frac{1}{T}\sum_{i}F_{i}\cdot J_{i}-\sum_{i}\nabla \left(\frac{\mu_{i}}{T}\right)\cdot J_{i}$$

(9) $$S_{ch}=\frac{1}{T}\sum_{k}\mu_{k}r_{k}$$

In the above-mentioned entropy generation rate, the proportion of entropy generation due to viscous dissipation in total entropy generation is extremely low and it is neglected in this work. Meanwhile, under the assumptions of neglecting the Dufour and Soret effects, external forces as well as the coupling phenomena, the local entropy generation rate due to other sub-processes can be calculated as [9]:

(10) $${S^{\prime \prime \prime }}_{c}=\frac{\lambda }{T^{2}}{\left(\frac{dT}{dx}\right)}^{2}$$

(11) $${S^{\prime \prime \prime }}_{diff}=\rho \sum_{k=1}^{N_{s}}\frac{D_{km}R_{u}}{X_{k}}\left(\frac{dY_{k}}{dx}\right)\left(\frac{dX_{k}}{dx}\right)$$

(12) $${S^{\prime \prime \prime }}_{ch}=-\frac{1}{T}\sum_{k=1}^{N_{s}}\sum_{i=1}^{N_{c}}\mu_{k}\omega_{i,k}$$

where λ is thermal conductivity, is the mass diffusivity of the *k*th species, $R_{u}$ is the universal gas constant, *X_k_* and *Y_k_* are mole fraction and mass fraction of *k*th species, $\mu_{k}$ is the chemical potential of *k*th species, and $\omega_{i,k}$ is the production rate of *k*th species and *i*th reaction.

### 2.3. Exergy Loss Rate

Furthermore, according to the Gouy–Stodola equation, the exergy loss due to the irreversibility of various processes can be obtained as [8]:

(13) $${e^{\prime \prime }}_{c}=T_{0}{S^{\prime \prime }}_{c},\;{e^{\prime \prime }}_{diff}=T_{0}{S^{\prime \prime }}_{diff},\;{e^{\prime \prime }}_{ch}=T_{0}{S^{\prime \prime }}_{ch}$$

where $T_{0}$ is the ambient temperature, and ${S^{\prime \prime }}_{c}$, ${S^{\prime \prime }}_{diff}$ and ${S^{\prime \prime }}_{ch}$ are total entropy generation due to heat conduction, mass diffusion, and chemical reactions, which can be obtained by integrating the local entropy generation along the volume.

Due to the incompleteness of the combustion process, the exergy carried by products of combustion process is unavailable in the energy utilization system. Therefore, it is also considered as exergy loss and can be calculated as follows [12]:

(14) $${e^{\prime \prime }}_{ex}=\dot{m}e_{ex}^{\mathrm{st}}$$

where $\dot{m}$ is the mass flow rate and $e_{ex}^{\mathrm{st}}$ is the standard chemical exergy of exhaust mixture after combustion. Therefore, the exergy loss ratio due to various reasons can be calculated as follows:

(15) $$E_{c}={e^{\prime \prime }}_{c}/{e^{\prime \prime }}_{F},\;E_{diff}={e^{\prime \prime }}_{diff}/{e^{\prime \prime }}_{F},\;E_{ch}={e^{\prime \prime }}_{ch}/{e^{\prime \prime }}_{F},\;E_{ex}={e^{\prime \prime }}_{ex}/{e^{\prime \prime }}_{F}$$

where ${e^{\prime \prime }}_{F}$ is exergy carried by the fuel and oxidant. It is worth noting that, due to the flow characteristics of the count-flow flame, a part of the fuel leaves the combustion area without participating in the combustion. Therefore, part of the fuel is not considered in the thermodynamics analysis.

## 3. Results

The mole fraction and velocity of fuel and oxidizer are shown in Table 1. As shown in Table 1, the mixtures of ammonia and ethylene, as well as argon and ethylene are considered fuels, in which the concentration of ammonia and argon is increased from 0 to 40%, respectively. The mixture of oxygen and nitrogen is considered an oxidant and the concentration of oxygen is set as 25%, while the inlet velocity of fuel and oxidizer is set as 16 cm/s. The ambient temperature and pressure are set as 298 K and 1 atm, respectively.

### 3.1. Exergy Loss Ratio Due to Heat Transfer, Mass Diffusion and Chemical Reaction

**Figure 1:**
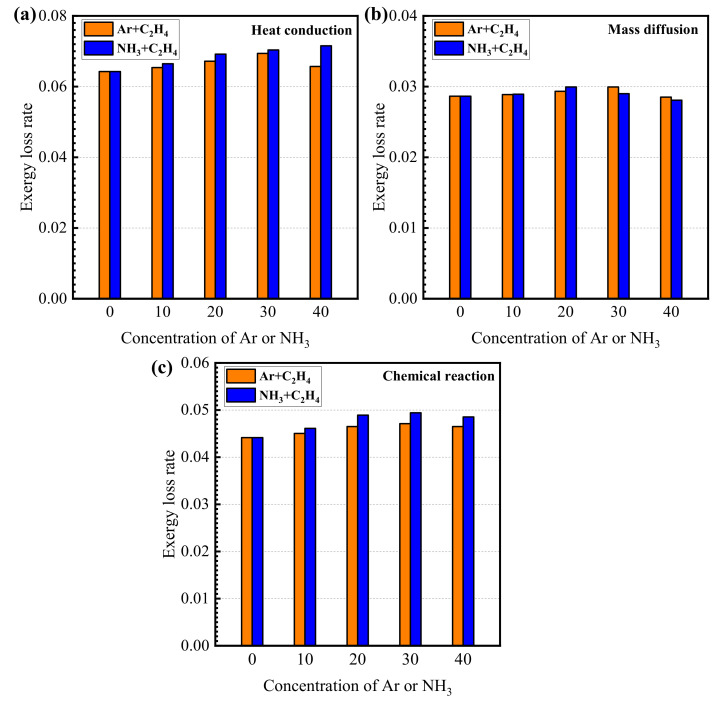
The variations in exergy loss rate due to irreversibility of (**a**) heat conduction, (**b**) mass diffusion and (**c**) chemical reaction with the concentrations of argon and ammonia in the fuel.

The variations in exergy loss rate due to the irreversibility of heat conduction with the concentrations of argon and ammonia in the fuel are shown in Figure 1a. According to Figure 1a, the exergy loss rate due to heat conduction in an ammonia/ethylene flame increases from 0.064 to 0.072 when the concentration of ammonia increases from 0 to 40%. For the argon/ethylene flame, the exergy loss rate due to heat conduction first increases and then decreases with the increase in the argon concentration in the fuel. When the argon concentration in the fuel is 30%, the exergy loss rate due to heat conduction reaches the highest value. Meanwhile, by comparing the cases of the ammonia/ethylene flame and the argon/ethylene flame, it can be seen that the exergy loss rate due to heat conduction in the ammonia/ethylene flame is higher than that of the argon/ethylene flame. The reason is that the ammonia addition increases the heat production and the thermal conductivity of the medium in the flame, which further enhances the heat conduction in the flame.

The variations in exergy loss rate due to the irreversibility of mass diffusion with the concentrations of argon and ammonia in the fuel are shown in Figure 1b. As shown in Figure 1b, the exergy loss rate due to mass diffusion is lower than that due to heat conduction and chemical reactions. In the argon/ethylene and ammonia/ethylene flames, as the concentrations of argon and ethylene in the fuel increase, the exergy loss rate due to mass diffusion has a trend of first increasing and then decreasing. The exergy loss rate due to mass diffusion reaches the maximum when the concentrations of argon and ammonia are 20% and 30%, respectively. Meanwhile, the ammonia addition has no obvious effect on the mass diffusion. When the ammonia concentration is high, the addition of ammonia reduces the mass diffusion process in the flame and causes a decrease in exergy loss rate due to mass diffusion.

The variations in exergy loss rate due to the chemical reaction with the concentrations of argon and ammonia in the fuel are shown in Figure 1c. According to Figure 1c, it can be seen that in the argon/ethylene flames and ammonia/ethylene flames, the exergy loss rate due to the chemical reaction increases with the increasing rate of the concentration of argon and ammonia. When the concentration of argon and ammonia reaches 20%, the influences of argon and ammonia in the fuel on the exergy loss rate due to the chemical reactions reduce. Meanwhile, it can be found that the exergy loss rate due to chemical traction of the ammonia/ethylene flame is much higher than that of the argon/ethylene flame, which indicates that the addition of ammonia has a significant enhancement on the chemical reactions in the flames.

### 3.2. Exergy Loss Rate Due to Incomplete Combustion 

**Figure 2:**
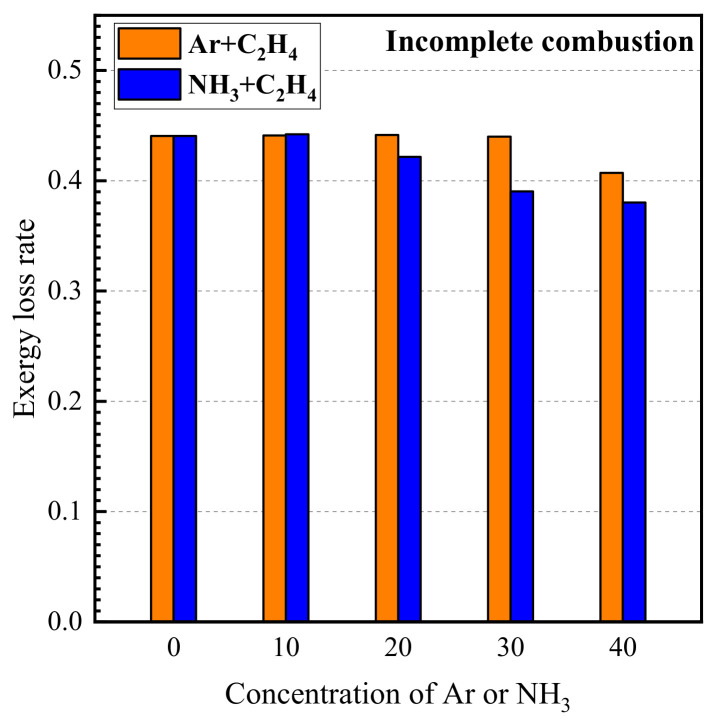
The variations in exergy loss rate due to incomplete combustion with the concentrations of argon and ammonia in the fuel.

The variation in exergy loss rate due to incomplete combustion with the concentrations of argon and ammonia in the fuel is shown in Figure 2. As shown in Figure 2, the exergy loss rate calculated in this work reached about 0.4. It is also much higher than the exergy loss due to the irreversibility of the combustion process. It indicates that, from the view of thermodynamics, incomplete flame combustion is a very important issue. Increasing the degree of combustion or realizing the re-utilization of exhausted gas can greatly improve the thermodynamic efficiency of the combustion process. Meanwhile, the exergy loss rate due to incomplete combustion decreases, and the degree of incomplete combustion of flame decreases when the concentration of argon and ammonia in the fuel increases from 10% to 40%. Comparing the results of argon/ethylene flame and ammonia/ethylene flame, it can be seen that the exergy loss due to incomplete combustion in ammonia/ethylene flame is higher than that of argon/ethylene flame. This indicates that the addition of ammonia reduces the degree of incomplete combustion.

**Figure 3:**
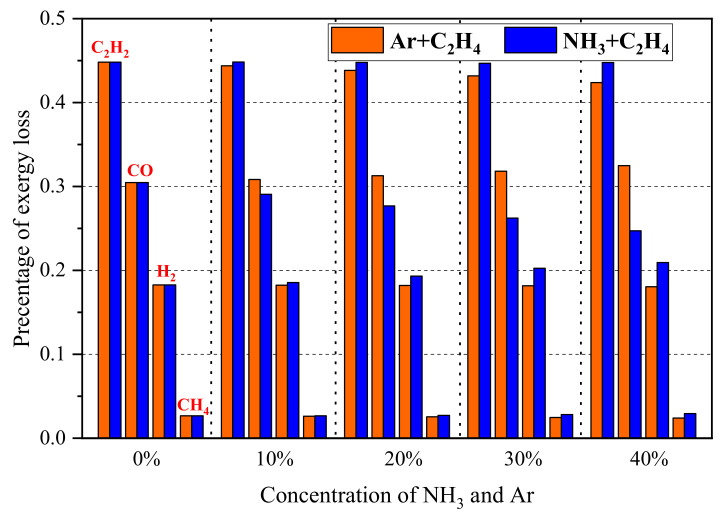
Proportions of exergy carried by each species in incomplete combustion products under different concentration of argon and ammonia.

In order to further study the exergy loss due to incomplete combustion, the proportions of exergy carried by each species in incomplete combustion products are calculated in this work, and these main sources are shown in Figure 3. The results reveal that the exergy carried by the three species, C_2_H_2_, CO, and H_2_, reaches more than 90% of total exergy loss due to incomplete combustion. The exergy carried by C_2_H_2_ is the highest, and it reaches more than 40% of total exergy loss due to incomplete combustion. Therefore, it can be foreseen that the exergy loss will decrease and thermodynamics efficiency will increase when considering these species as a circulating fuel. We calculated the exergy loss rate of pure ethylene flame considering C_2_H_2_, CO, and H_2_ as the circulating gas, and the result reveals that, when the exergy carried by C_2_H_2_, CO, and H_2_ is re-utilized, the total exergy loss rate of flame reduces by about 31%, 20%, and 15%, respectively. Additionally, the proportion of exergy carried by CO decreases, and that of H_2_ increases with an increase in the concentration of NH_3_ in fuel. This is because the CO addition decreases and H_2_ increases with the increase in concentration of ammonia.

### 3.3. Total Exergy Loss Rate and the Lowest Emission of Pollutants 

**Figure 4:**
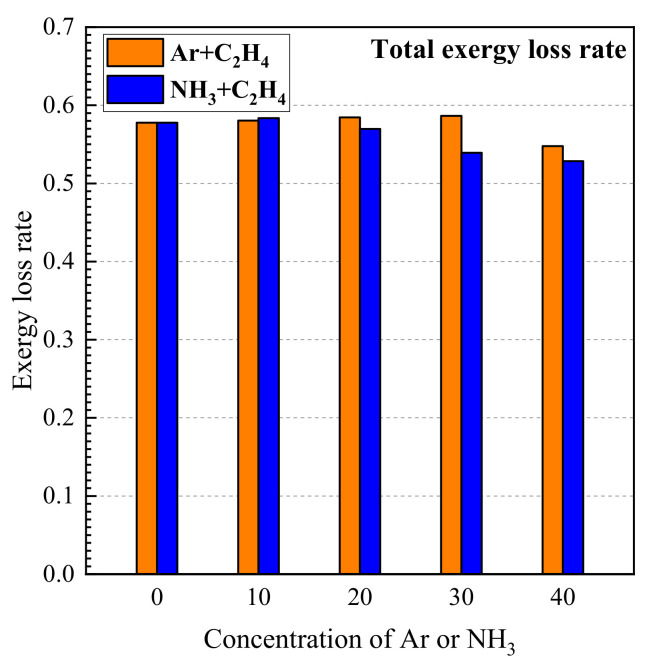
The variations in exergy loss rate due to incomplete combustion with the concentrations of argon and ammonia in the fuel.

The variations in exergy loss rate due to a chemical reaction with the concentrations of argon and ammonia in the fuel are shown in Figure 4. As shown in Figure 4, more than 50% of exergy is lost in the argon/ethylene flame and in the ammonia/ethylene flame. Meanwhile, the total exergy loss rate has a tendency of increasing first and then decreasing with an increase in the concentration of argon and ammonia. The total exergy loss rate reaches the maximum when the argon concentration is 10% and the ammonia concentration is 30% in the argon/ethylene flame and in the ammonia flame, respectively. Additionally, it is worth noting that, when the ammonia concentration in the fuel exceeds 20%, the influence of the ammonia addition on the total exergy loss rate is more obvious, and that the ammonia/ethylene flame is lower than the argon/ethylene flame. This indicates that the ammonia addition improves the thermodynamics efficiency of the combustion process.

In order to analyze the influence of the addition of ammonia on NOx and CO_2_ emissions, we proposed a parameter named the lowest emission of pollutants (LEP) based on thermodynamic analysis in this work. It establishes the relationship between exergy loss and the emission of pollutants in the combustion process and indicates the lowest emission of pollutants toward the environment when one-joule available work is obtained by a combustion process. Furthermore, the LEP can be calculated as:

(16) $$\mathrm{LEP}=\frac{Q_{pol}}{e_{F}\left(1-E\right)}$$

where $Q_{pol}$ is the total gas flow rate leaving the combustion zone. It can be obtained as follows based on count-flow flames considered in the work:

(17) $$Q_{pol}=2\int_{0}^{L}a_{l}\cdot X_{l}dl/V_{m}$$

where *a* is the ratio of radial velocity of gas and radius, *X* is the mole fraction of the pollutant, *L* is the distance between the fuel nozzle and the oxidant nozzle. *V_m_* is the molar volume of the gas, which is mainly affected by the gas temperature. 

**Figure 5:**
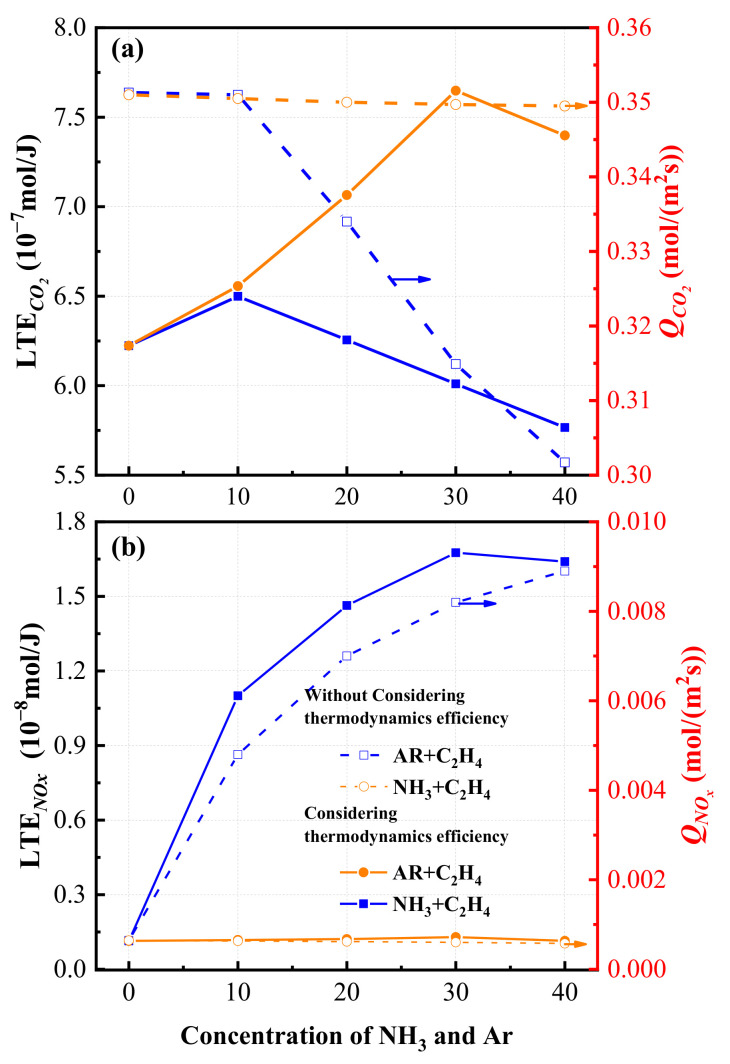
The variations in lowest thermodynamic emission value of (**a**) CO_2_ and (**b**) NOx with the concentrations of argon and ammonia.

The variations in lowest emission of NOx (LEP_NOx_) and CO_2_ (LEP_CO2_) with the concentrations of argon and ammonia in the fuel and total flow rate of CO_2_ (*Q*_NOx_) and NOx (*Q*_CO2_) are shown in Figure 5. As shown in Figure 5, it can be seen that both the LEP_CO2_ and *Q*_CO2_ and in argon/ethylene flame are significantly higher than that in the ammonia/ethylene flame. This indicates that the addition of ammonia can effectively reduce the emission of CO_2_. However, the tendencies of LEP_CO2_ and *Q*_CO2_ are different, in which *Q*_CO2_ decreases and LEP_CO2_ shows a trend of first increasing and then decreasing with an increase in argon and ammonia concentrations. The LEP_CO2_ reaches the maximum when the ammonia concentration is 10% and the Ar concentration is 30%. Additionally, in terms of NOx emissions, it can be seen that due to the presence of nitrogen in the oxidant, the argon/ethylene gas will release a small amount of NOx. Meanwhile, according to the LEP_NOx_ and *Q*_NOx_ of the ammonia/ethylene flame, the parameter LEP also reflects information differently with gas flow rate *Q*. The increasing tendency of LEP_NOx_ weakens gradually when the ammonia concentration increases, and it even decreases slightly when the ammonia concentration in the fuel increases from 30% to 40%. *Q*_NOx_ increases with the increase in ammonia concentration. The reason is that the thermal efficiency of the combustion process increases with the increasing level of ammonia, which also reduces fuel consumption and pollutant emissions.

### 3.4. Chemical Reaction Paths Analysis Based on Thermodynamics Analysis

**Figure 6:**
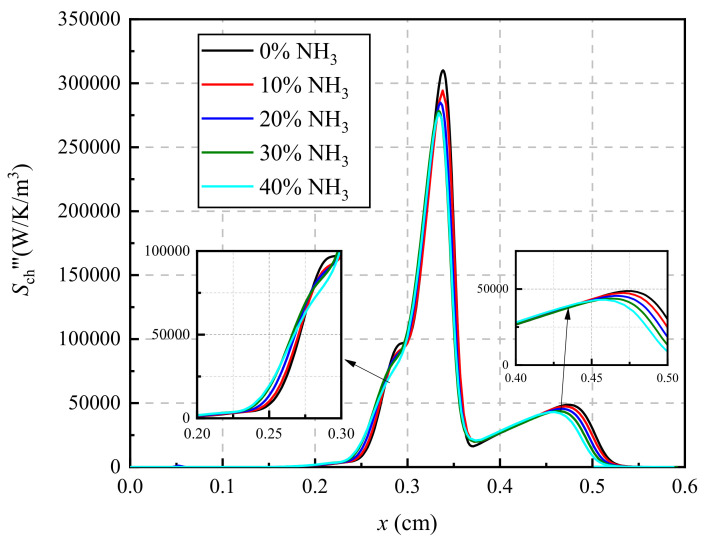
The local entropy generation rate due to chemical reaction.

From the perspective of thermodynamics, the chemical potential is the cause of the chemical reaction, which determines the direction and intensity of the chemical reaction [8], and chemical entropy generation reflects the overall change of chemical potential in each chemical reaction. Therefore, the analysis of chemical entropy generation is helpful for the chemical reaction path analysis, and the identification of important chemical reactions is of great interest to us. The variation in local entropy generation rates due to a chemical reaction with ammonia concentration in fuel is shown in Figure 6. As shown in Figure 6, the local entropy generation rate distribution exists in two peaks, which are located near 0.3 cm and 0.45 cm, respectively. With the increase in the ammonia concentration in the fuel, the peaks of the local chemical entropy generation rate distribution gradually move toward the fuel nozzle. Meanwhile, the peak values of local chemical entropy generation rate near 0.33 cm and that near 0.45 cm decreases when the ammonia concentration in the fuel increases from 0 to 40%. In addition, it is noted that the local chemical entropy generation has a significant increase in the region of 0.25 cm to 0.3 cm.

**Figure 7:**
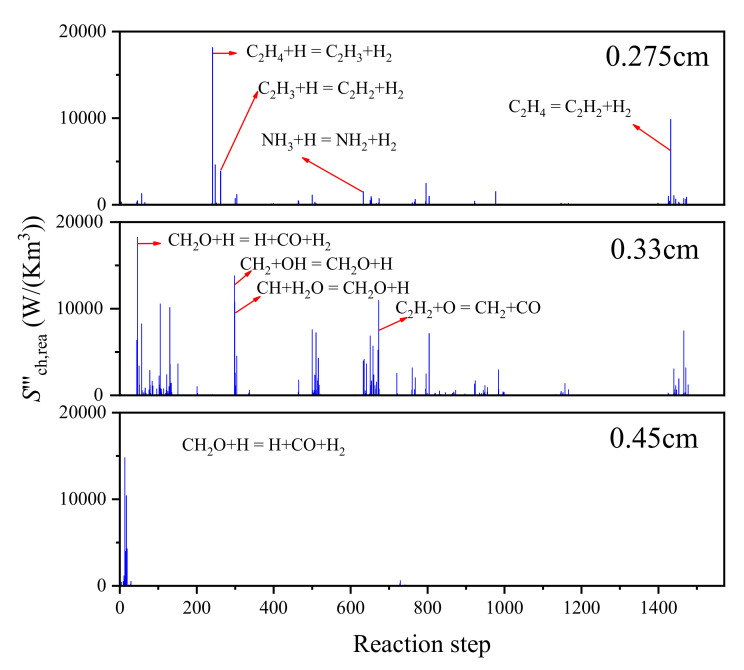
The local entropy generation rate due to chemical reaction.

To explain the local chemical entropy generation rate distribution, it is essential to determine the key substances and chemical reaction paths that relate to entropy generation and exergy loss. When the ammonia concentration is 40%, chemical entropy generation rates due to each step of the reaction in the region of 0.275 cm, 0.33 cm, and 0.45 cm are calculated, and these are shown in Figure 7. In general, the chemical reaction in the ammonia/ethylene flame can be divided into three parts. The first part is pyrolysis reactions of fuel, which occurs in the region of 0.25 cm to 0.5 cm and can be divided into two groups of reactions.

The pyrolysis reactions of hydrocarbon, such as:

(R1) $$\begin{array}{l} C_{2}H_{4}+{H=C}_{2}H_{3}{+H}_{2}\\ C_{2}H_{3}+{H=C}_{2}H_{2}{+H}_{2} \end{array}$$

The pyrolysis reactions of ammonia, such as:

(R2) $${\mathrm{NH}}_{3}+{H=\mathrm{NH}}_{2}{+H}_{2}$$

Meanwhile, it is worth noting that the entropy generation rates due to pyrolysis reactions of ethylene are much higher than that of ammonia. It accounts for 80% of the total local chemical entropy generation rates.

The second part is the chemical reactions between pyrolysis products, as well as the formation of PAHs, which occurs in the region near 0.33 cm. The chemical reaction in this stage is relatively complicated. However, it is not difficult to find some key chemical reactions and substances. The four reactions that produce the highest chemical entropy production are shown as follows. From R3 to R5, it can be found that CH_2_O is present in the reactants and products. This also shows that CH_2_O is very important for the chemical reaction in this stage.

(R3) $${\mathrm{CH}}_{2}{O+H=H+\mathrm{CO}+H}_{2}$$

(R4) $${\mathrm{CH}}_{2}+{\mathrm{OH}=\mathrm{CH}}_{2}O+H$$

(R5) $${\mathrm{CH}+H}_{2}{O=\mathrm{CH}}_{2}O+H$$

(R6) $$C_{2}H_{2}{+O=\mathrm{CH}}_{2}+\mathrm{CO}$$

The third part is the oxidation of elementary substances including H atoms, O atoms, and OH, which occurs in the region near 0.45 cm. The chemical entropy generation in this region is mainly from three chemical reactions, which are shown as follows:

(R7) $${H+O}_{2}{=\mathrm{HO}}_{2}$$

(R8) $${\mathrm{HO}}_{2}+{O=O}_{2}+\mathrm{OH}$$

(R9) $${\mathrm{HO}}_{2}+{\mathrm{OH}=O}_{2}{+H}_{2}O$$

## 4. Conclusions

The exergy analysis of counter-flow diffusion flames with ammonia/ethylene and argon/ethylene binary fuels is presented in this work. Exergy loss and exergy loss rate are calculated and the effects of ammonia on exergy loss rate due to heat conduction, mass diffusion, and chemical reaction are analyzed. The exergy loss rate due to incomplete combustion and the total exergy loss rate are also given. Some other works are also carried out based on the thermodynamics analysis. A parameter, the lowest emission of pollutants, was proposed to evaluate CO_2_ and NOx emissions of the different combustion processes. The chemical reaction paths in ammonia/ethylene flames are analyzed, and important chemical reactions and substances are identified. The main conclusions are given as follows:

(1) The exergy loss rate due to heat conduction and exergy loss rate due to chemical reaction increase with the increase in ammonia concentration. The exergy loss rate due to the mass diffusion process has a trend of first increasing and then decreasing with the increase in ammonia concentration. The ammonia addition has a significant enhancement on heat transfer and chemical reaction in the flames, but it has no obvious effect on the mass diffusion.
(2) Incomplete combustion is an important cause for the production of exergy loss and C_2_H_2_, CO, and H_2_ are the main species for exergy loss due to incomplete combustion. The exergy loss rate due to incomplete combustion decreases with the increase in the concentration of argon and ammonia in the fuel.
(3) Total exergy loss rate has a tendency of first increasing slightly and then decreasing with an increase in the concentration of ammonia. When ammonia concentration is high, ammonia addition decreases the total exergy loss rate and enhances the thermodynamics effectivity of combustion obviously. A parameter, the lowest emission of pollutants, is proposed based on thermodynamic analysis. The results indicate that ammonia addition can effectively reduce the emission of CO_2_. When ammonia concentration increases, the increasing tendency of LEP_NOx_ weakens gradually and it reaches the maximum when ammonia concentration is 30%.
(4) The chemical reactions in the ammonia/ethylene flame can be divided into three parts. These are the pyrolysis process of fuel, chemical reactions between pyrolysis products, as well as the oxidation of elementary substances. The results reveal that CH_2_O is recognized as an important substance in the chemical reactions.

## Figures and Tables

**Table 1:** The species and mole fraction of fuel.

Ar/C_2_H_4_ Flame		NH_3_/C_2_H_4_ Flame	
*X*_C2H4_	*X*_AR_	*X*_C2H4_	*X*_NH3_
1	0	1	0
0.9	0.1	0.9	0.1
0.8	0.2	0.8	0.2
0.7	0.3	0.7	0.3
0.6	0.4	0.6	0.4

## Data Availability

All data included in this study are available upon request by contacting the corresponding author.
